# Macrophage Migration Inhibitory Factor and Stearoyl-CoA Desaturase 1: Potential Prognostic Markers for Soft Tissue Sarcomas Based on Bioinformatics Analyses

**DOI:** 10.1371/journal.pone.0078250

**Published:** 2013-10-22

**Authors:** Hiro Takahashi, Robert Nakayama, Shuhei Hayashi, Takeshi Nemoto, Yasuyuki Murase, Koji Nomura, Teruyoshi Takahashi, Kenji Kubo, Shigetaka Marui, Koji Yasuhara, Tetsuro Nakamura, Takuya Sueo, Anna Takahashi, Kaname Tsutsumiuchi, Tsutomu Ohta, Akira Kawai, Shintaro Sugita, Shinjiro Yamamoto, Takeshi Kobayashi, Hiroyuki Honda, Teruhiko Yoshida, Tadashi Hasegawa

**Affiliations:** 1 Graduate School of Horticulture, Chiba University, Matsudo, Chiba, Japan; 2 Graduate School of Bioscience and Biotechnology, Chubu University, Kasugai, Aichi, Japan; 3 Plant Biology Research Center, Chubu University, Kasugai, Aichi, Japan; 4 Division of Genetics, National Cancer Center Research Institute, Tokyo, Japan; 5 Cancer Transcriptome Project, National Cancer Center Research Institute, Tokyo, Japan; 6 Department of Orthopaedic Surgery, Keio University School of Medicine, Tokyo, Japan; 7 Department of Applied Life Science, Faculty of Biotechnology and Life Science, Sojo University, Kumamoto, Japan; 8 Department of Dermatology, Tokyo Medical and Dental University, Tokyo, Japan; 9 Department of Biotechnology, School of Engineering, Nagoya University, Nagoya, Aichi, Japan; 10 Orthopedics Division, National Cancer Center Hospital, Tokyo, Japan; 11 Department of Surgical Pathology, Sapporo Medical University School of Medicine, Sapporo, Hokkaido, Japan; 12 Pathology Division, National Cancer Center Hospital, Tokyo, Japan,; University of Virginia, United States of America

## Abstract

The diagnosis and treatment of soft tissue sarcomas (STSs) has been particularly difficult, because STSs are a group of highly heterogeneous tumors in terms of histopathology, histological grade, and primary site. Recent advances in genome technologies have provided an excellent opportunity to determine the complete biological characteristics of neoplastic tissues, resulting in improved diagnosis, treatment selection, and investigation of therapeutic targets. We had previously developed a novel bioinformatics method for marker gene selection and applied this method to gene expression data from STS patients. This previous analysis revealed that the extracted gene combination of *macrophage* migration inhibitory factor (MIF) and stearoyl-CoA desaturase 1 (SCD1) is an effective diagnostic marker to discriminate between subtypes of STSs with highly different outcomes. In the present study, we hypothesize that the combination of *MIF* and *SCD1* is also a prognostic marker for the overall outcome of STSs. To prove this hypothesis, we first analyzed microarray data from 88 STS patients and their outcomes. Our results show that the survival rates for *MIF*- and *SCD1*-positive groups were lower than those for negative groups, and the *p* values of the log-rank test are 0.0146 and 0.00606, respectively. In addition, survival rates are more significantly different (*p* = 0.000116) between groups that are double-positive and double-negative for *MIF* and *SCD1*. Furthermore, *in vitro* cell growth inhibition experiments by MIF and SCD1 inhibitors support the hypothesis. These results suggest that the gene set is useful as a prognostic marker associated with tumor progression.

## Introduction

Soft tissue sarcomas (STSs) are a group of highly heterogeneous tumors that exhibit a diverse spectrum of mesenchymal differentiations. In addition, the molecular diagnosis of tumor heterogeneity has been hampered by the relatively low incidence of these tumors; an annual incidence of soft tissue sarcoma is around 50 per million population[1,2] , i.e. <1% of all malignant tumors. Significant differences were observed in 5-year survival rates among STS subtypes, e.g., 100% for well-differentiated liposarcoma (WLS), 71% for synovial sarcoma (SS), 46% for pleomorphic malignant fibrous histiocytoma (MFH), and 92% for myxofibrosarcoma (MFS). Recently, MFH is frequently called “undifferentiated pleomorphic sarcoma (UPS)”, because it was renamed as such in 2002 by the World Health Organization (WHO) [3]. The primary objective of molecular diagnosis is to identify a set of marker genes that facilitates accurate differential diagnosis of sarcoma subtypes. Discrimination between MFH and MFS, for example, is particularly difficult because there is a histological overlap between the two and because of the high heterogeneity of MFH [4]. Information on such subtype-specific genes may also help in understanding the molecular pathways that are activated in each subtype of the different biological malignancies. To this end, we had previously developed the projective adaptive resonance theory (PART) filtering method [5] for gene filtering and the boosted fuzzy classifier with SWEEP operator (BFCS) method [6,7] for model construction. Further, we developed a combination of these method, termed PART-BFCS [8-12], and applied this method to analyze gene expression data from STSs [12]. Our previous analysis showed that the 28 extracted genes are useful markers and that the most frequently selected combination of genes, *macrophage* migration inhibitory factor (MIF) and stearoyl-CoA desaturase 1 (SCD1), represents an effective diagnostic marker combination to discriminate between MFH and MFS.

In general, the objective of a statistical or informatical analysis is the enrichment of important information [13-18]. The use of statistical or informatical analysis is both practical and useful [19-28]. In the present study, we hypothesized that the combination of *MIF* and *SCD1* can serve not only a diagnostic marker for discrimination between MFH and MFS, but also act as a prognostic marker for the overall outcome of STS, since an elevated expression of *MIF* and *SCD1* is observed in various highly malignant tumors [29-32]. Accordingly, using clinical and microarray data from STS patients, we conducted a simulation based on the permutation test, to extract genes that have both the functions of “a diagnostic marker to discriminate between MFH and MFS” and “a prognostic marker for the outcome of overall STS”, which yielded four statistically significant genes, including *MIF* and *SCD1*. Furthermore, we investigated the potential of the combination of *MIF* and *SCD1* as a prognostic marker using clinical and microarray data from STS patients. We also investigated the *in vitro* cell growth inhibition induced by MIF and SCD1 inhibitors in a mouse MFH cell line. These experiments suggest that the combination of *MIF* and *SCD1* is useful as a prognostic marker for tumor progression.

## Materials and Methods

### Ethics statement

The study was conducted according to the principles expressed in the Declaration of Helsinki, and the ethics committee of the National Cancer Center approved the study protocol and all patients provided written informed consent to participate.

### Patients and tumor samples

Characteristics of the 88 soft tissue tumors used in this study are shown in Table S1. All patients had received a histological diagnosis of primary soft tissue tumor at National Cancer Center Hospital, Tokyo, between 1996 and 2002. Tumor samples were obtained at the time of excision and cryopreserved in liquid nitrogen until use. 

### Microarray analysis

For RNA extraction, trained pathologists carefully excised the tissue samples from the main tumor, leaving a margin clear from the surrounding non-tumorous tissue. Microscopically, the samples may still contain several non-tumor cells. Elimination of non-tumor stromal cells is important for gene expression analyses in carcinomas since tumor tissues contain a significant number of non-tumor stromal cells, including fibroblasts, endothelial cells, and inflammatory cells. Otherwise, tumor tissues contain very few non-tumor stromal cells for soft tissue sarcomas. Furthermore, soft tissue sarcomas contain non-tumor stromal cells, which are difficult to eliminate since soft tissue sarcoma originates from mesenchymal cells. Therefore, laser microdissection was not performed in this study. Total RNAs extracted from the bulk tissue samples were biotin-labeled and hybridized to high-density oligonucleotide microarrays (Human Genome U133A 2.0 Array, Affymetrix, Santa Clara, CA, USA) comprising 22,283 probe sets representing 18,400 transcripts, according to the manufacturer's instructions. The scanned array data were processed by Affymetrix Microarray Suite v.5.1 (MAS5), which scaled the average intensity of all the genes on each array to the target signal of 1,000. The microarray data in the present paper are available from the Genome Medicine Database of Japan (GeMDBJ) (https://gemdbj.nibio.go.jp/dgdb/) under the accession number EXPR058P.

### Data processing

We excluded 68 controls and 2,343 genes that were subject to cross-hybridization according to NetAffx Annotation (www.affymetrix.com). Furthermore, we excluded those genes for which more than 10 percent (8/88) of the samples showed an absent call (i.e., the detection call determined by MAS5 based on the *p* value of the one-sided Wilcoxon signed-rank test; absent call corresponds to *p* ≥ 0.065, which is the default threshold in MAS5), since this indicates that the expression signal was undetectable. We also excluded those genes that showed an interquartile range value of less than 2,000. In total, 1,376 genes were selected, to which we applied log-transformation or binarization using the median values as the threshold for each gene. The two types of datasets, binarized and log-transformed, were used for Welch's *t*-test and the log-rank test, respectively. 

### Simulation on the basis of permutation test

**Figure 1:**
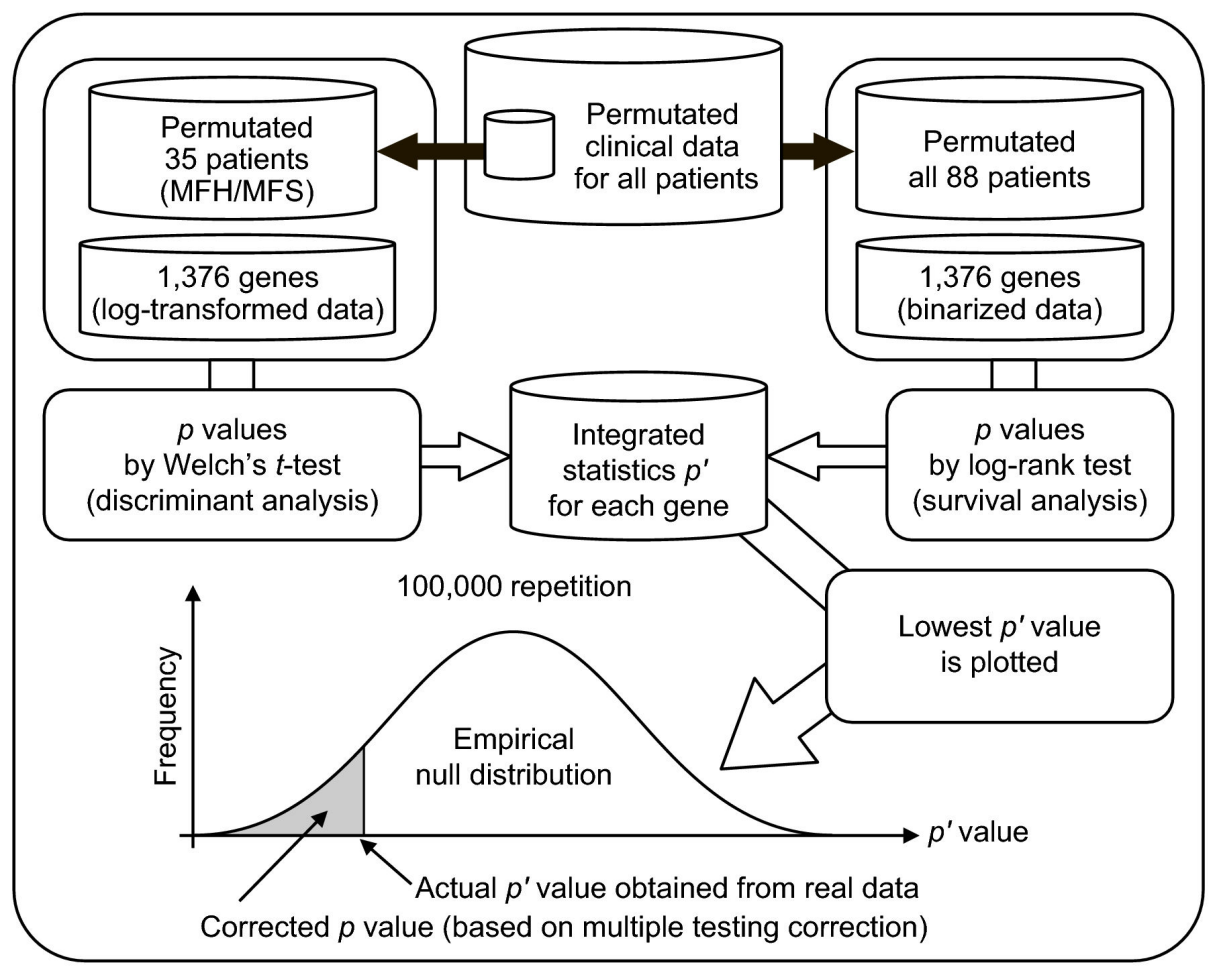
A schematic of the simulation conducted based on the permutation test. Clinical data for all patients were permutated. Permutated data for 35 STS patients (20 MFH patients and 15 MFS patients) were extracted from permutated data for all patients. For these data, *p* values (*p*_1_) were calculated by applying Welch's *t*-test to the log-transformed gene expression data to discriminate between MFH and MFS. Otherwise, permutated data for 88 patients were used for survival analysis. For these data, *p* values (*p*_2_) were calculated by applying log-rank test to the binarized gene expression data to analyze the outcome in the STS patient group. The integrated statistic *p*' was defined as *p*_1_ × *p*_2_. The lowest *p*' value was selected for each repetition. This procedure was repeated 100,000 times, and an empirical null distribution was constructed. Using the distribution, the actual *p*' value obtained from real data was converted to the corrected *p* value (based on the correction for multiple testing).

We calculated *p* values by applying Welch's *t*-test for discrimination between MFH and MFS, and by applying the log-rank test in the survival analysis of all STS patients for the 1,376 filtered genes. We defined the integrated statistic *p*' as *p*_1_ × *p*_2_, where *p*_1_ indicates the *p* value calculated by Welch's *t*-test and *p*_2_ indicates the *p* value calculated by the log-rank test. STS patients (n = 35; 20 MFH patients and 15 MFS patients) were used in both these tests, and the outcomes between MFH and MFS were significantly different. We therefore conducted a simulation based on the permutation test as shown in Figure 1 to estimate the corrected *p* values to address this problem, as well as the multiple testing problems. 

### Statistical analysis

Median values of gene expression signals for each gene were calculated and patients were divided into 2 groups using the median values as the threshold for each gene. Log-rank tests [33] were conducted for the outcome of STS patients for each gene, and Spearman's rank correlation coefficients for relationships between histological grades (or tumor metastases) and gene expression signals were also calculated. In the present study, we analyzed over 50 months of follow-up information as censored data. Kaplan-Meier curves [34] were drawn for all STS patients, and those positive for *MIF*, *SCD1*, and the combination of *MIF* and *SCD1*.

### Cell lines and culture conditions

Murine sarcoma Sendai (MuSS) [35], a malignant fibrous histiocytoma cell line, was provided by the Cell Resource Center for Biomedical Research Institute of Development, Aging and Cancer, Tohoku University. Cells were maintained in RPMI 1640 medium containing 2% fetal bovine serum (FBS) and 1% penicillin-streptomycin solution. Cell cultures were incubated at 37°C, 5% CO_2_, and 100% humidity. 4-Iodo-6-phenylpyrimidine (4-IPP) (purchased from TOCRIS Bioscience, Bristol, UK) and A939572 (purchased from Biofine International, Vancouver, BC, Canada) were dissolved in dimethyl sulfoxide (DMSO).

### Cell growth assays

For the determination of concentrations for the combination of 4-IPP (MIF inhibitor) and A939572 (SCD1 inhibitor), cells were plated in 96-well plates (10,000 cells per well). After 24 h of incubation, cells were treated with 4-IPP at concentrations ranging from 5µM to 50 µM, A939572 at concentrations ranging from 1 nM to 1000 nM, a combination of both, or DMSO only (control). After 24 h of incubation of cells with these compounds, cell viability was measured using the 3-(4,5-dimethylthiazol-2-yl)-2,5-diphenyl-tetrazolium bromide (MTT) assay [36]. Metabolically active cells can convert the yellow tetrazolium salt MTT to indigo blue formazan. For the assay, the medium were removed gently and the cells were washed with PBS. Subsequently, medium containing 0.5 g/L MTT was added to each well. After 2 h of incubation of the cells with MTT, the medium was removed gently and the cells were washed with PBS. To dissolve the indigo blue formazan crystals, 100µL DMSO was added. The optical density was measured on a microplate reader at 540 nm. All *in vitro* studies were repeated 3 times.

## Results

### Discrimination between MFH and MFS by using gene expression levels of *MIF* and *SCD1*

**Figure 2:**
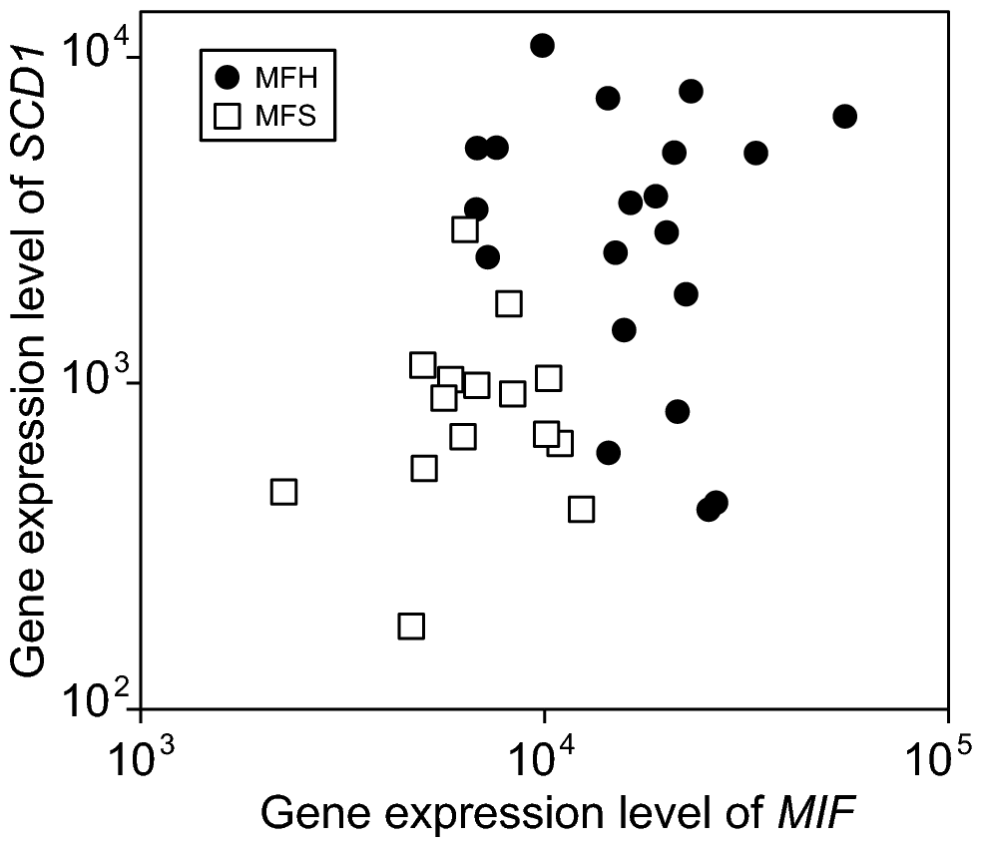
Discrimination between MFH and MFS by using expressions of *MIF* and *SCD1*. Open square indicates MFS patient. Filled circle indicates MFH patient.

Our previous analysis revealed that *MIF* and *SCD1* is an effective diagnostic marker to discriminate between MFH and MFS [12]. In the present study, we first analyzed microarray data from 35 STS patients (20 MFH patients and 15 MFS patients) by using expressions of *MIF* and *SCD1*, as shown in Figure 2. Figure 2 shows that STS patients are clearly classified into two groups (MFS and MFS). 

### Extraction of genes with both diagnostic and prognostic marker functions by simulation on the basis of the permutation test

In the present study, we hypothesized that the combination of *MIF* and *SCD1* is useful not only a diagnostic marker for discrimination between MFH and MFS, but also represents a prognostic marker for the overall outcome of STS. Therefore, we first conducted a simulation based on a permutation test for the outcome of overall STS, from which four genes, including *MIF* and *SCD1*, were extracted (Table 1). From this analysis, it can be seen that the corrected *p* values are 0.00523 and 0.0198 for *MIF* and *SCD1*, respectively. This result suggests that *MIF* and *SCD1* have functions of both a diagnostic marker to discriminate between MFH and MFS and prognostic marker for the overall outcome of STS.

**Table 1:** Genes extracted by simulation based on the permutation test.

**Affymetrix**	**Accession**	**Gene**	***P* value**		**Integrated**	**Corrected**
**probe ID**	**no.**	**symbol**	***t*-test**	**Log-rank test**	**statistics *p*'**	***p* value**
207543_s_at	NM_000917	*P4HA1*	1.22E-04	5.73E-04	7.01E-08	0.00487
217871_s_at	NM_002415	*MIF*	5.31E-06	1.46E-02	7.75E-08	0.00523
201231_s_at	NM_001428	*ENO1*	1.06E-04	1.06E-03	1.12E-07	0.00693
200832_s_at	AB032261	*SCD1*	7.36E-05	6.06E-03	4.46E-07	0.01976

### Association analysis of clinical and gene expression data

We next conducted a log-rank tests of STS patient outcomes and a correlation analysis between tumor metastasis and each clinical parameter, as shown in Tables 2 and 3, respectively. Table 2 shows that only the histological grade is significantly associated with patient outcomes (*p* = 0.00102 as calculated by the log-rank test). Table 3 shows that histological grade is strongly correlated with incidence of tumor metastasis in STS patients (ρ = 0.486, *p* = 1.56 × 10^-6^ as calculated by the Spearman's rank correlation coefficient). The histological grade is a useful conventional index, and pathologists trained in the detection of specific tumors can diagnose these indices in patients. Therefore, it is important to identify factors that correlate with histological grade and patient outcomes for the diagnosis of STS patients. We conducted log-rank tests of STS patient outcomes and a correlation analysis of the histological grade or incidence of tumor metastasis with the expression of four genes selected by the permutation test, as shown in Table 4. Table 4 shows that *MIF* has a high correlation with histological grade (ρ = 0.421, *p* = 4.41 × 10^-5^) and tumor metastasis (ρ = 0.308, *p* = 3.47 × 10^-3^), whereas *SCD1* has no correlation with histological grade (ρ = -0.0191, *p* = 0.860) and tumor metastasis (ρ = 0.0237, *p* = 0.826). This result suggests that *MIF* expression is strongly related with tumor metastasis, whereas *SCD1* expression can be used in combination with the histological grade to diagnose the STS outcome.

**Table 2:** Relationships between clinical parameters and outcome of STS patients.

**Clinical parameters**		**Number of patients**	***p* value of log-rank test**
Gender	Male	46	0.652
	Female	42	
Age	< 55	45	0.173
	≥55	43	
Tissue-type	MLS	20	0.0670
	MFH	20	
	MFS	15	
	SS	17	
	LMS	6	
	FS	5	
	MPNST	5	
Histological grade	1	14	0.00102
	2	23	
	3	51	

MLS: Myxoid liposarcoma, MFH: Pleomorphic malignant fibrous histiocytoma, MFS: Myxofibrosarcoma, SS: Synovial sarcoma, LMS: Leiomyosarcoma, FS: Fibrosarcoma, MPNST: Malignant peripheral nerve sheath tumor.

**Table 3:** Correlation between clinical parameters and tumor metastasis of STS patients.

**Clinical parameters**		**Number of patients**	**Spearman's rank correlation coefficient**	
			***ρ***	***p* value**
Gender	Male	46	-0.206	0.0543
	Female	42		
Age		88	0.0327	0.762
Histological grade	1	14	0.486	1.56E-06
	2	23		
	3	51		

**Table 4:** Correlation analysis using Spearman's rank correlation coefficient.

**Affymetrix probe ID**	**Accession no.**	**Gene symbol**	**Spearman's rank correlation coefficient**			
			**with histological grade**		**with tumor metastasis**	
			***ρ***	***p* value**	***ρ***	***p* value**
207543_s_at	NM_000917	*P4HA1*	0.449	1.12E-05	0.424	3.89E-05
217871_s_at	NM_002415	*MIF*	0.421	4.41E-05	0.308	3.47E-03
201231_s_at	NM_001428	*ENO1*	0.356	6.66E-04	0.247	2.01E-02
200832_s_at	AB032261	*SCD1*	-0.0191	0.860	0.0237	0.826

### Analysis of the outcomes of STS patients using gene expression data

**Figure 3:**
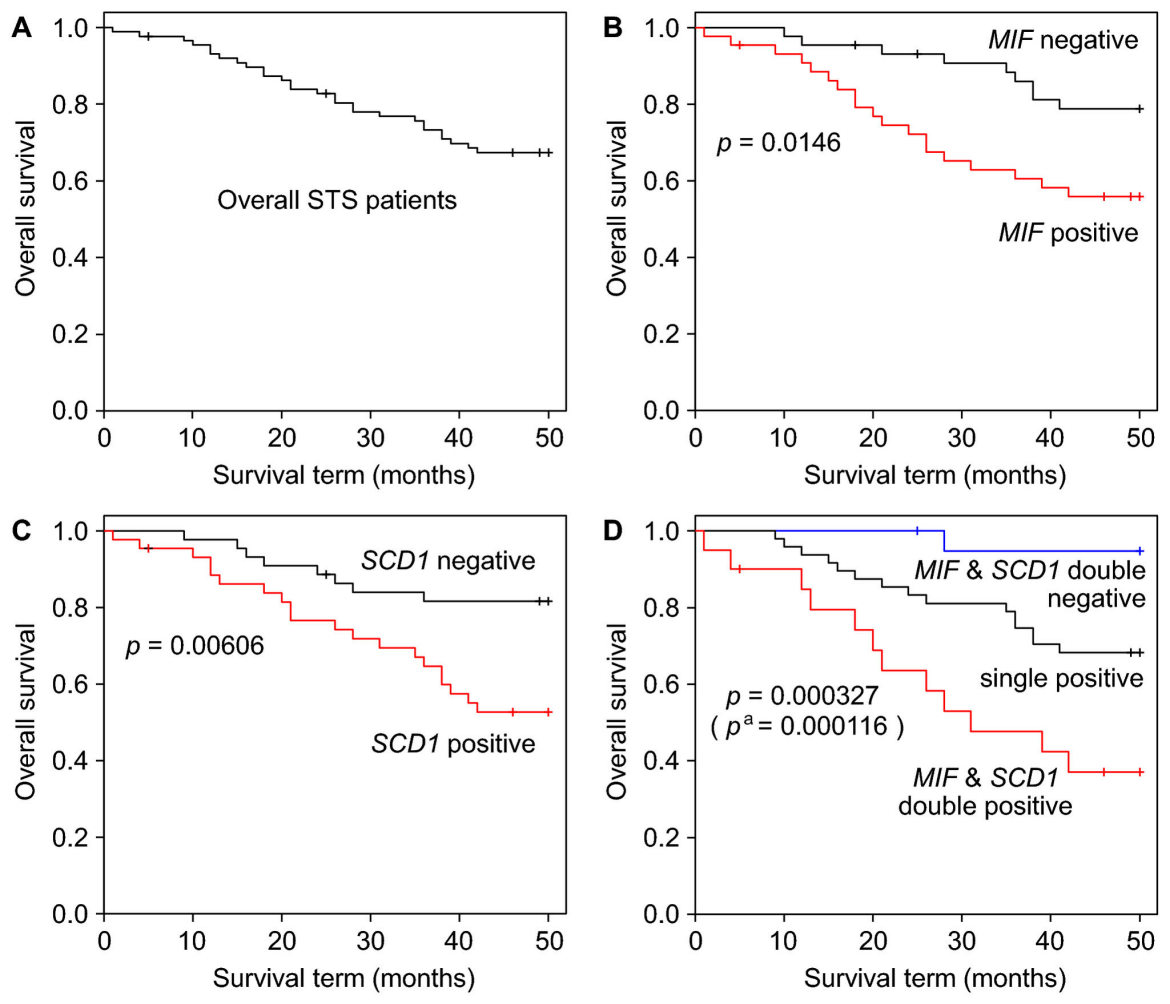
Kaplan-Meier curves and log-rank test for all STS patients. (**A**) All STS patients (**B**) The *MIF*-positive group (*MIF* > 10171, median of *MIF* signals for 88 patients) consisted of 44 patients (red line) and the *MIF*-negative group consisted of 44 patients (black line). (**C**) The *SCD1*-positive group (SCD1 > 1879, median of *SCD1* signals for 88 patients) consisted of 44 patients (red line) and of *SCD1*-negative group consisted of 44 patients (black line). (**D**) The *MIF* and *SCD1* double-positive group (*MIF* > 10171 and SCD1 > 1879) consisted of 20 patients (red line), the *MIF* or *SCD1* single-positive group consisted of 48 patients (black line), and the *MIF* and *SCD1* double-negative group consisted of 20 patients (blue line). The *p* values were calculated by the log-rank test. ^a^ indicates the *p* value for the comparison of the double-positive *vs*. the double-negative groups.

Patients were divided into 2 groups using the median values as the threshold for each gene for binarization of independent variable. For example, the median values were 10,171 and 1,879 for *MIF* and *SCD1*, respectively. The interquartile range values (difference in median values between positive and negative groups) were 10,142 and 2,681 for *MIF* and *SCD1*, respectively. We conducted log-rank tests of STS patient outcomes by using binarized data. In the present study, we focused on *MIF* and *SCD1* to test the hypothesis that the combination of *MIF* and *SCD1* could be a prognostic marker for the overall outcome of STSs. Thus, Kaplan-Meier curves were drawn for all STS patients, and those positive for *MIF*, *SCD1*, and the combination of *MIF* and *SCD1*, as shown in Figure 3. Figure 3B shows that the survival rate of the *MIF*-positive group (n = 44) was significantly lower than that of the *MIF*-negative group (n = 44) (*p* = 0.0146 as calculated by the log-rank test). Similarly, Figure 3C shows that the survival rate of the *SCD1* positive group (n = 44) was significantly lower than that of the *SCD1*-negative group (n = 44) (*p* = 0.00606 as calculated by the log-rank test). Furthermore, Figure 3D shows that the survival rates among groups that are double-positive (n = 20), single-positive (n = 48), and double-negative (n = 20) for *MIF* and *SCD1* are significantly different (*p* = 0.000327 as calculated by the log-rank test), and that the survival rate of the double-positive group (n = 20) was much lower than that of the double-negative group (n = 20) with greater significance (*p* = 0.000116 as calculated by the log-rank test). These results indicate that *MIF*, *SCD1*, and the combination of *MIF* and *SCD1* are not only diagnostic markers to discriminate between MFH and MFS, but also potential prognostic markers for the overall outcome of STS. Furthermore, the combination of *MIF* and *SCD1* is superior to the single markers, *MIF* or *SCD1*, as a prognostic marker. 

### Survival analysis for each tissue type using a combination of *MIF* and *SCD1*

**Figure 4:**
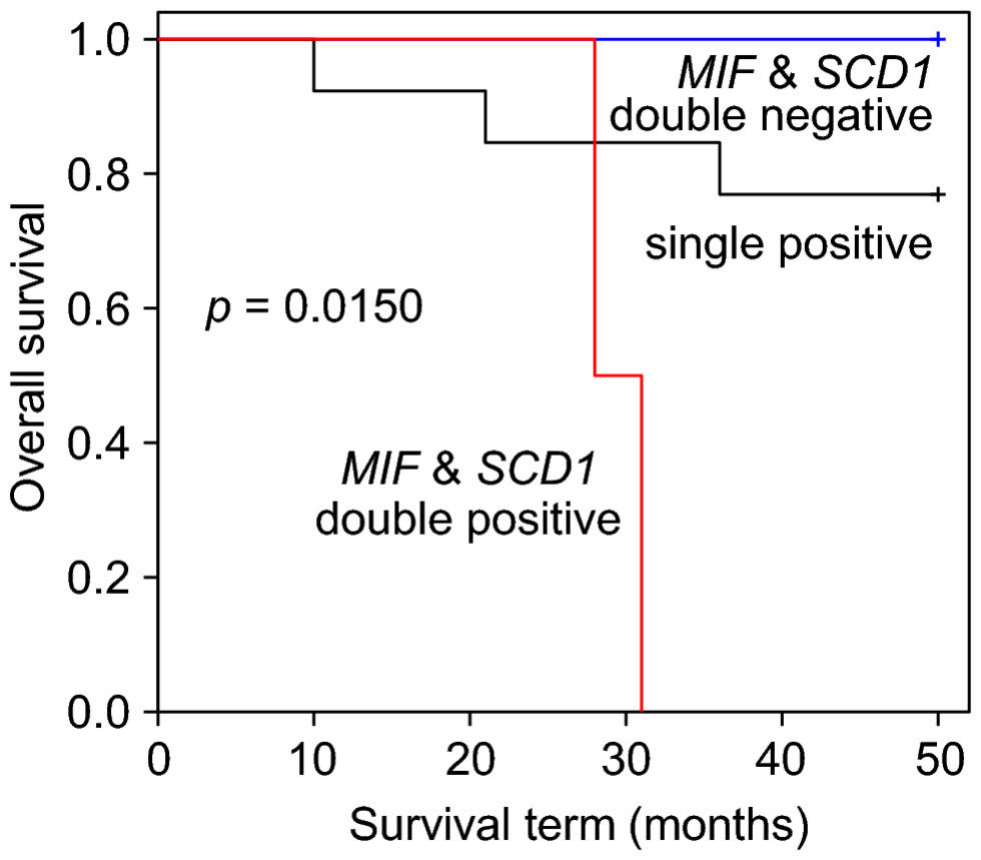
The Kaplan-Meier curve and log-rank test for MLS patients. The *MIF* and *SCD1* double-positive group (*MIF* > 10171 and SCD1 > 1879) consisted of 2 patients (red line), the *MIF* or *SCD1* single-positive group consisted of 13 patients (black line), and the *MIF* and *SCD1* double-negative group consisted of 5 patients (blue line). The *p* value was calculated by the log-rank test.

The differential diagnosis of soft tissue sarcomas is not difficult, with the exception of discrimination between MFH and MFS. We conducted a survival analysis by classifying patients into one of three groups based on a combination of *MIF* and *SCD1* expression for each tissue type, as shown in Table 5. The survival rates among groups that are double-positive (n = 2), single-positive (n = 13), and double-negative (n = 5) for *MIF* and *SCD1* are significantly different (*p* = 0.0150 as calculated by the log-rank test) for MLS patients, as shown in Figure 4 and Table 5. This result suggests that a combination of *MIF* and *SCD1* is a potential prognostic maker for MLS. The number of patients in each category is relatively small however, and these results should be validated in future studies.

**Table 5:** Survival analysis for each tissue type using a combination of *MIF* and *SCD1*.

**Tissue-type**	**Number of patients**	***P* value of log-rank test**
MLS	20	0.0150
MFH	20	0.868
MFS	15	0.638
SS	17	0.231
LMS	6	0.638
FS	5	0.0634
MPNST	5	0.886

MLS: Myxoid liposarcoma, MFH: Pleomorphic malignant fibrous histiocytoma, MFS: Myxofibrosarcoma, SS: Synovial sarcoma, LMS: Leiomyosarcoma, FS: Fibrosarcoma, MPNST: Malignant peripheral nerve sheath tumor.

### Cell growth inhibition assay using MIF inhibitor and SCD1 inhibitor

**Figure 5:**
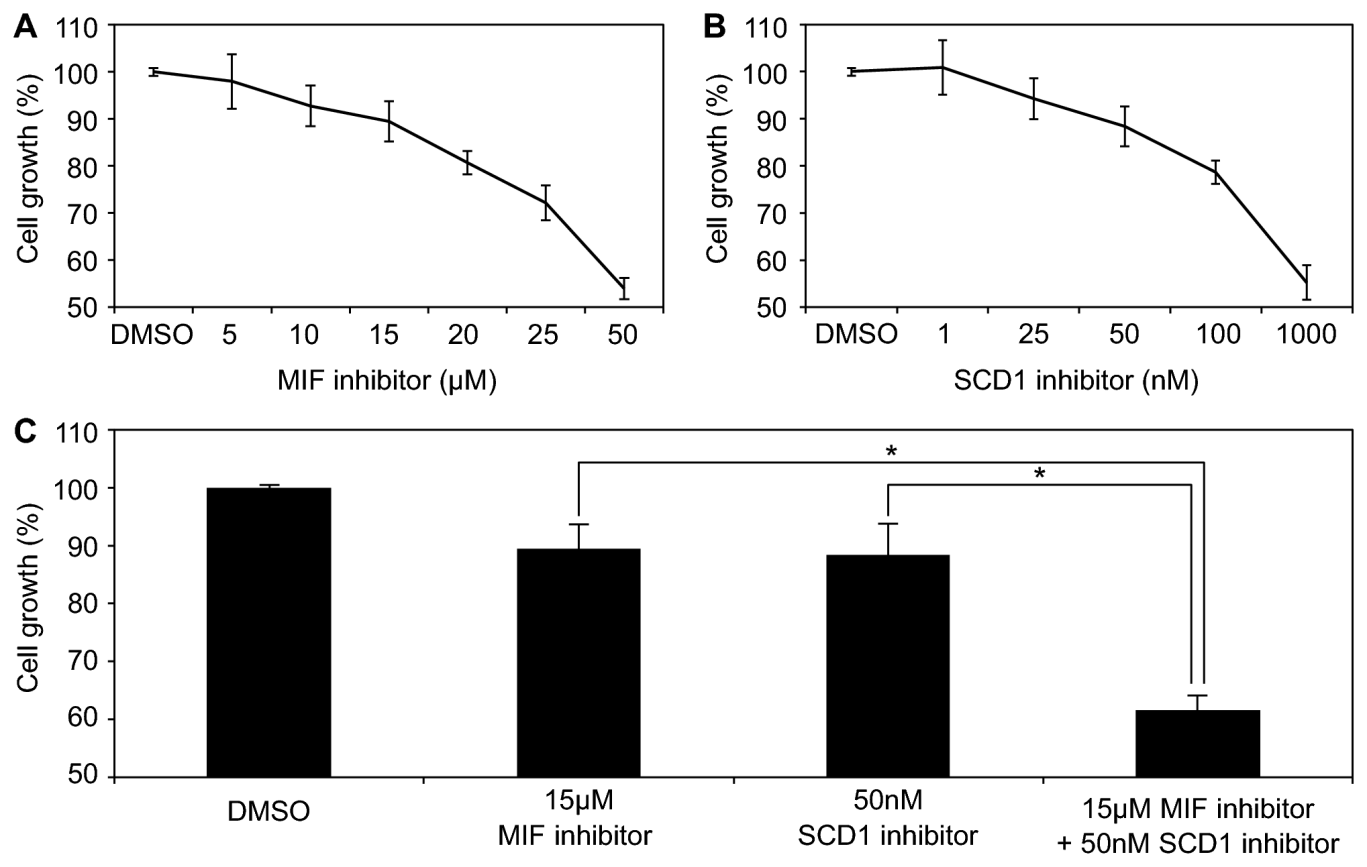
Cell growth inhibitory effects of 4-IPP and A939572 for MuSS cell line. (**A**) Inhibition of cell viability at varying concentrations of 4-IPP (MIF inhibitor) in MuSS cells relative to DMSO-treated control cells. (**B**) Inhibition of cell viability at varying concentrations of A939572 (SCD1 inhibitor) in MuSS cells relative to DMSO-treated control cells. (**C**) Inhibition of cell viability by DMSO only (control), 15 µM MIF inhibitor, 50 nM SCD1 inhibitor, and the combination of 15 µM MIF inhibitor and 50 nM SCD1 inhibitor in MuSS cells relative to DMSO-treated control cells. Data are shown for cells treated for 24 h in media containing 2% FBS. Cell viability was determined using the MTT assay in 3 independent replicates at each dose level. Error bars represent the SD from the mean. * indicates *p* < 0.05 (*p* value was calculated by Welch’s *t*-test and corrected by Bonferroni correction for multiple testing).

We conducted cell growth inhibitory experiments using the MuSS cell line with a MIF inhibitor and a SCD1 inhibitor to investigate the role of MIF, SCD1, and the combination of MIF and SCD1 for tumor progression. In the present study, 4-IPP and A939572 were used as MIF and SCD1 inhibitors, respectively. The effects of 4-IPP, A939572, and the combination of 4-IPP and A939572 on cell proliferation were evaluated in a 24-hour growth inhibition assay, as shown in Figure 5. Figure 5A and 5B show concentration response curves for cells treated with 4-IPP and A939572, respectively. This is clearly dose-dependent. Furthermore, we selected 15 µM 4-IPP and 50 nM A939572 as concentrations that showed weak cell growth inhibition (approximately 10%) to evaluate the inhibitory effect of the combination of inhibitors on MuSS cells. Figure 5C shows that cell growth was significantly inhibited to a greater extent by the combination of inhibitors than by the single inhibitors. This result was statistically significant (*p* < 0.05 as calculated by the Welch's *t*-test and corrected by the Bonferroni correction). These results indicate that MIF and SCD1 are potential essential factors of cell growth for STSs.

## Discussion

In the present study, we conducted the simulation based on the permutation test for the extraction of genes that have both functions of the diagnostic marker to discriminate between MFH and MFS and the prognostic marker for the outcome of overall STS. Consequently, only four genes, including *MIF* and *SCD1*, were extracted and their corrected *p* values were statistically significant (*p* < 0.05). We focused on the combination of *MIF* and *SCD1*.

MIF was first reported in the 1960s as a factor capable of inhibiting the random migration of macrophages during delayed-type hypersensitivity responses [37-39]. Recently, studies have been conducted on the roles of MIF in various inflammatory diseases, such as rheumatoid arthritis [40] and atherosclerosis [41]. (S,R)-3-(4-hydroxyphenyl)-4,5-dihydro-5-isoxazole acetic acid methyl ester (ISO-1), which has been used for the treatment of various inflammatory diseases, was reported to be an inhibitor of the biological activity of MIF [42]. Subsequent to our report[12], *in vitro* MIF inhibition experiments have been conducted using small molecules for various cancers, such as colon cancer inhibited by ISO-1 [43], prostate cancer inhibited by ISO-1 [44], lung cancer inhibited by ISO-1 [45], and 4-Iodo-6-phenylpyrimidine (4-IPP) [45], glioblastoma inhibited by ISO-1[46-48], and adenoid cystic carcinoma inhibited by ISO-1[49]. In addition, several experiments have been conducted on animal models (*in vivo*) such as those for colorectal cancer inhibited by ISO-1 [50], and prostate cancer inhibited by ISO-1 [44]. As mentioned, MIF inhibition is an effective treatment method for various tumors. Although MIF inhibition suppresses both cell growth and metastasis in many tumors, its inhibition of cell growth is not universal. For example, MIF depletion or pharmacologic inhibition in breast tumor cells changed tumor growth only slightly, but blocked metastasis, as reported by Simpson et al [51].

SCD catalyzes the conversion of saturated fatty acids (SFAs) to ∆9 monounsaturated fatty acids (MUFAs). These enzymes preferentially convert stearic acid (C18:0) to oleic acid (C18:1) and palmitic acid (C16:0) to palmitoleic acid (C16:1)[52]. In humans, 2 genes have been characterized (*SCD1* and *SCD5*), *SCD1* being co-orthologous to the 4 mice genes [53]. SFAs and MUFAs, the most abundant fatty acid species, have many divergent biological effects including the regulation of cell proliferation, programmed cell death, and lipid-mediated cytotoxicity [54]. Recently, a number of reports have implicated *SCD1* expression and activity in the pathogenesis of cancer [55]. Overexpression of human *SCD1* was observed in a variety of human cancers, including colon, esophageal, and hepatocellular carcinomas relative to the corresponding normal tissues [30]. Subsequent to our report[12], *in vitro* SCD1 inhibition experiments were conducted using small molecules for several cancers, such as lung cancer and breast cancer inhibited by N-(2-(6-(3,4-dichlorobenzylamino)-2-(4-​methoxyphenyl)-3-oxopyrido[2,3-b]pyrazin​-4(3H)-yl)ethyl)acetamide (CVT-11127) [56-58], pharyngeal cancer and lung cancer inhibited by 4-(2-chlorophenoxy)-N-(3-(3-methylcarbamoyl)phenyl)piperidine-1-carboxamide (A939572) [59], and colon cancer inhibited by 5-tetradecyloxy-2-furoic acid (TOFA) [60]. In addition, a single *in vivo* study has been conducted using a human gastric cancer xenograft model that was inhibited by A939572 [59]. As mentioned, SCD1 inhibition is an effective treatment method for several tumors.

 In the present study, cell growth arrest for STS cells was observed by experiments using MIF and SCD1 inhibitors, as shown in Figure 5. However, the cancer cell growth assay is not the first report of the use of MIF or SCD1 inhibitors to retard cancer cell growth. MIF or SCD1 inhibitors have been used to restrict the growth or metastasis of many types of cancers [43-48,56-60], although not STS. Moreover, gene silencing of MIF or SCD1 has been carried out for many kinds of cancers [44,48,54,59-67], again with the exception of STS. Although MIF- or SCD1 inhibition has not been reported in the case of STS, and our assay is insufficient to show the effects of MIF- or SCD1-inhibition for STS, many previous studies have suggested that MIF and SCD1 are potential therapeutic targets in the treatment of STS patients. Our association analysis, which examines overall outcome and metastasis in STS patients, also supports this hypothesis. 

**Figure 6:**
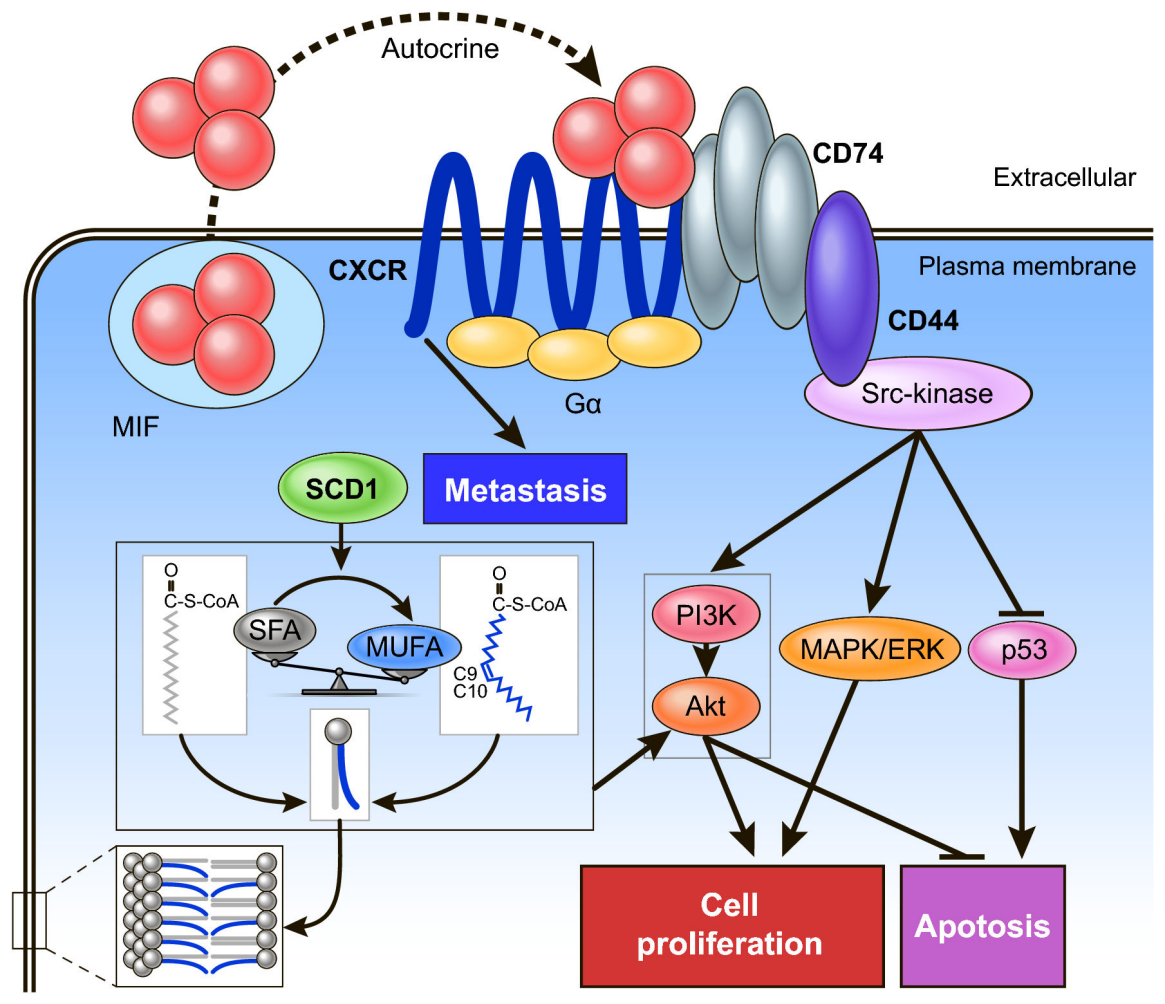
Hypothetical regulation model for metabolic and signaling control by MIF and SCD1. MUFA, monounsaturated fatty acids; SFA, saturated fatty acids; SCD1, stearoyl-CoA desaturase 1; MIF, macrophage migration inhibitory factor; CXCR, CXC chemokine receptor; PI3K, phosphoinositide 3-kinase; MAPK, extracellular signal-regulated kinase; ERK, mitogen-activated protein kinase.

 Although we did not conduct experiments to identify the molecular mechanism of action of MIF- and SCD1 inhibitors, several studies examining MIF and SCD1 pathways have been reported to date, as shown in Figure 6. Secreted MIF interacts with cell surface CD74 [68]. CD74 lacks a signal-transducing intracellular domain but interacts with the proteoglycan CD44 and mediates signaling via CD44 to induce the activation of a Src-family kinase and mitogen-activated protein kinase (MAPK)/extracellular signal-regulated kinase (ERK), to activate the phosphatidylinositol 3-kinase (PI3K)/Akt pathway, or to initiate p53-dependent inhibition of apoptosis [69]. MIF also can induce invasion and metastasis via G-protein–coupled chemokine receptors (CXCR2 and CXCR4) [43,70]. While the SCD1 enzyme activates the Akt pathway by regulation of the MUFA/SFA balance in mammalian cell lipids [55,71]. As mentioned above, both MIF and SCD1 regulate cell viability through various pathways and there is a common signaling pathway downstream of MIF and SCD1. Furthermore, MIF is related with incidence of tumor metastasis in patients. They are therefore reasonable potential therapeutic targets. Combination chemotherapy may prevent drug resistance in cancer patients. However, further studies, such as analysis of the molecular mechanism, invasion, apoptosis assays, and other experiments should be conducted for STS cells in the future.

 MIF inhibitors have been developed as anti-inflammatory drugs and some anti-inflammatory drugs have a MIF inhibitory effect. For example, Ibudilast (current development codes: AV-411 or MN-166) is an anti-inflammatory drug that was initially developed for the treatment of bronchial asthma and inhibits MIF activity [72]. Safety evaluation for Ibudilast has been conducted by a clinical trial [73]. Furthermore, the occurrence of MIF inhibitors in cruciferous vegetables as a natural product [74] suggests that MIF will be more a promising therapeutic target in the future.　On the other hand, SCD1 inhibitors have been developed as drugs for diabetes and dyslipidemia [75]. Studies have been conducted to establish the therapeutic window for the SCD1 inhibitor MK-8245 using various animals, such as mouse, rat, dog, and monkey [75]. Therefore, MIF and SCD1 inhibitors have high potential as drugs, because the safety of these inhibitors has been validated for various diseases other than cancer, as mentioned above. Although a differential diagnosis between MFH and MFS is difficult even for trained pathologists. Our bioinformatics analysis has shown the combination of *MIF* and *SCD1* is a useful diagnostic marker, and can hence be useful in this context. Furthermore, our analysis has demonstrated that the utility of *MIF* and *SCD1* is not limited to discrimination between MFH and MFS. Specifically, *MIF* expression correlates to the incidence of metastasis and histological grade in all STS patients, and *SCD1* expression provides novel information in addition to histological grade, which would help in determining the prognosis of STS.

In conclusion, we conducted exhaustive gene expression analysis of STS patients and *in vitro* growth inhibition assays using an MFH cell line to test the hypothesis that the gene combination of *MIF* and *SCD1* (identified by a bioinformatics approach) is not only a diagnostic marker for discrimination between MFH and MFS, but also a prognostic marker for the overall outcome of STS. Our results from these experiments suggest that the combination of *MIF* and *SCD1* is potentially useful as a prognostic marker associated with tumor progression or metastasis in patients.

## Supporting Information

Table S1 **Clinical data of 88 STS patients.**
(XLS)
